# Supplementary material for: Optimising PHBV biopolymer production in haloarchaea via CRISPRi-mediated redirection of carbon flux
Source: Commun Biol. 2021 Aug 25;4:1007. doi: 10.1038/s42003-021-02541-z (PMC8387396; doi:10.1038/s42003-021-02541-z)
Supplement: Supplementary file 2 — Supplementary Information [file 42003_2021_2541_MOESM2_ESM.pdf]

Supplementary information

Optimising PHBV biopolymer production in haloarchaea *via* CRISPRi-mediated redirection of carbon flux

Lin Lin[1,2], Junyu Chen[1], Ruchira Mitra[1,3], Quanxiu Gao[1], Feiyue Cheng[1,2], Tong Xu[1], Zhenqiang Zuo[1], Hua Xiang[1,2,*] & Jing Han[1,2,*]

Affiliations

[1] State Key Laboratory of Microbial Resources, Institute of Microbiology, Chinese Academy of Sciences, Beijing 100101, People's Republic of China

[2] College of Life Science, University of Chinese Academy of Sciences, 100049, Beijing, People's Republic of China

[3] International College, University of Chinese Academy of Sciences, 100049, Beijing, People's Republic of China

Supplementary Methods

CR-RT-PCR (circularized RNA reverse transcription PCR)

The circularized RNA reverse transcription PCR (CR-RT-PCR) was used to identify the transcription start site (TSS) as previously described[1]. The cDNA was prepared by reverse transcription of self-ligated RNA with random hexamer primers (Thermo Fisher Scientific, USA), which was used as the PCR template. The PCR products amplified with the primer pairs CR-XX-F/R (Table S2) were cloned into the pMD18-T vector to determine the TSS by DNA sequencing.

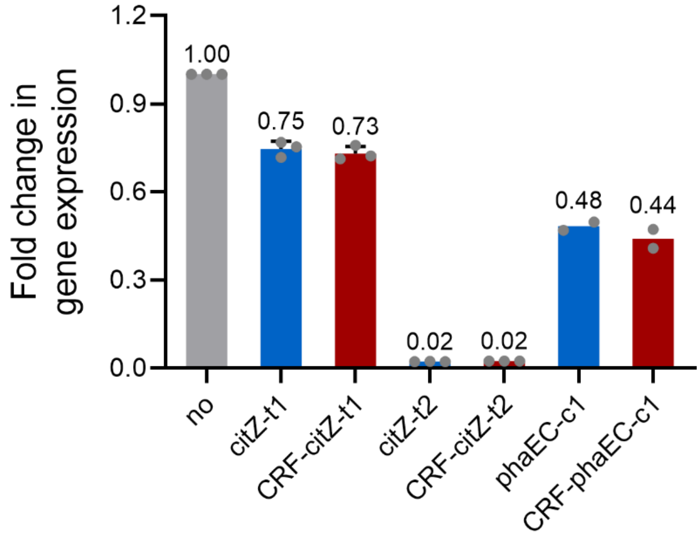

Supplementary Fig. 1. The repression effects produced by three crRNAs before and after the knockout of CRISPR arrays. The gray column represents the control with no crRNA expression. The blue columns represent the inhibition before knockout of CRISPR arrays, and the red columns represent the inhibition after knock outing CRISPR arrays (CRF). Data shown for two or three biological replicates. Error bars indicate SDs, n = 3.

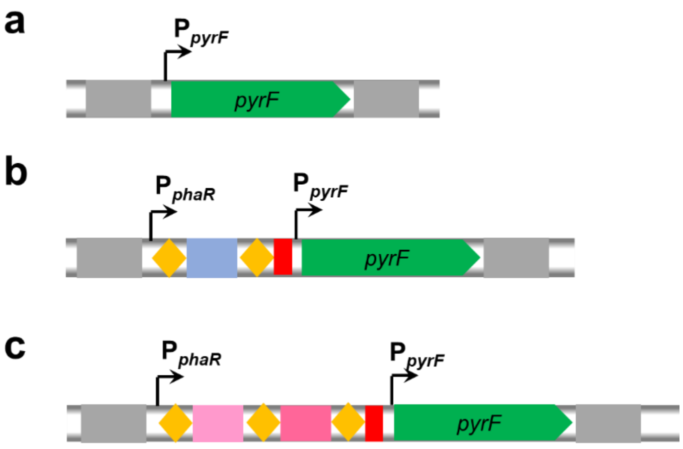

Supplementary Fig. 2. Chromosomal integration of crRNA expression cassette at the original locus of *pyrF* gene in the genome. a The inserted fragment of *pyrF* gene. **b** The inserted fragment of *pyrF* gene and mini-CRISPR structure for expressing NT. **c** The inserted fragment of *pyrF* gene and mini-CRISPR structure for expressing citZ-t2 and gltA-t1. The transcription of crRNA and *pyrF* gene are driven by P_{phaR} and P_{pyrF}, respectively. Repeats, orange diamonds; NT spacer, light blue; citZ -t2, light pink; gltA-t1, pink; T8 terminator: red rectangle; Upstream and downstream fragments of *pyrF*, grey rectangles.

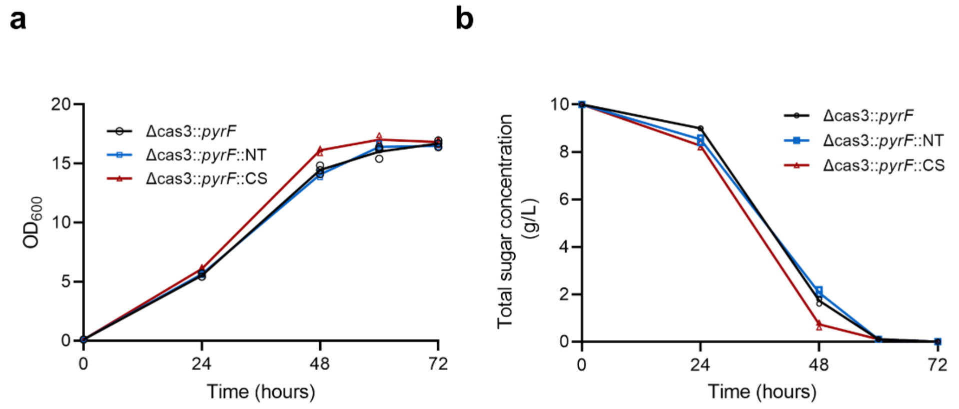

Supplementary Fig. 3. *citZ* repression on cell growth and glucose consumption of *H. mediterranei via* CRISPRi by chromosomal integration crRNA expression system. **a** Cell growth. **b** Glucose consumption. Data shown for three biological replicates.

Supplementary Table 1. PHBV accumulation by *H. mediterranei* strains with citrate synthase genes repressed *via* CRISPRi by chromosomal integration crRNA expression system[a].

Strains	CDW (g/L)[b]	PHBV content (%)[c]	PHBV concentration (g/L)	3HV fraction (mol%)	PHBV productivity (g/L·d)
DF50ΔEPSΔ *cas3::pyrF*	9.02 ± 0.39	43.11 ± 1.39	3.88 ± 0.08	11.20 ± 0.48	1.29 ± 0.03
DF50ΔEPSΔ *cas3::pyrF::* NT	8.91 ± 0.20	43.12 ± 2.69	3.84 ± 0.19	10.70 ± 0.36	1.28 ± 0.06
DF50ΔEPSΔ *cas3::pyrF::* CS	9.16 ± 0.03	45.08 ± 1.18	4.13 ± 0.12	10.58 ± 0.37	1.38 ± 0.04

[a]All data are expressed as mean ± standard deviations from three independent experiments and strains were cultivated in MG medium for 3 days. [b]CDW, dry weight of the cell (in grams) produced per liter of culture. [c]PHBV content, the weight percent of PHBV in CDW.

Supplementary Table 2. Strains used in this study.

Strains	Relevant characteristics	Source or reference
E. coli JM109	*recA1 supE44 endA1 hsdR17 gyrA96 relA1 thi*	2
E. coli JM110	*dam dcm* mutant of *E. coli* JM109	3
H. mediterranei DF50ΔEPS	*pyrF* and HFX_2145-2148 deletion mutant of *H. mediterranei*	4
H. mediterranei 50BΔ2549	P_{phaR} insertion and HFX_2549 deletion mutant of *H. mediterranei* DF50ΔEPS	5
H. mediterranei CRF	CRISPR-free mutant of *H. mediterranei* DF50	6
H. mediterranei DF50ΔEPSΔ*cas3*	*cas3* deletion mutant of *H. mediterranei* DF50ΔEPS	This study
H. mediterranei 50BΔ2549Δ*cas3*	*cas3* deletion mutant of *H. mediterranei* 50BΔ2549	This study
H. mediterranei CRFΔEPSΔ*cas3*	HFX_2145-2148 and *cas3* deletion mutant of *H. mediterranei* CRF	This study
H. mediterranei DF50ΔEPSΔ*cas3::pyrF*	*pyrF* complementation mutant of DF50ΔEPSΔ*cas3*	This study
H. mediterranei DF50ΔEPSΔ*cas3::pyrF::*NT	*pyrF* complementation mutant and mini-CRISPR (NT) insertion mutant of DF50ΔEPSΔ*cas3*	This study
H. mediterranei DF50ΔEPSΔ*cas3::pyrF::*CS	*pyrF* complementation mutant and mini-CRISPR (CS) insertion mutant of DF50ΔEPSΔ*cas3*	This study

Supplementary References

1. Kuhn, J. RT-PCR analysis of 5' to 3'-end-ligated mRNAs identifies the extremities of *cox2* transcripts in pea mitochondria. Nucleic Acids Res. 30, 439-446 (2002).
2. Sambrook, J., Fritsch, E. F. & Maniatis, T. Molecular cloning: a laboratory manual, 2nd ed. Cold Spring Harbor Laboratory Press, Cold Spring Harbor, NY (1989).
3. Palmer, B. R. & Marinus, M. G. The *dam* and *dcm* strains of *Escherichia coli*--a review. Gene. 143, 1-12 (1994).
4. Zhao, D. H. et al. Improving polyhydroxyalkanoate production by knocking out the genes involved in exopolysaccharide biosynthesis in *Haloferax mediterranei*. Appl. Microbiol. Biotechnol. 97, 3027-3036 (2013).
5. Zuo, Z. Q. et al. Engineering *Haloferax mediterranei* as an efficient platform for high level production of lycopene. Front. Microbiol. 9 (2018).
6. Li, M. et al. Characterization of CRISPR RNA biogenesis and Cas6 cleavage-mediated inhibition of a provirus in the haloarchaeon *Haloferax mediterranei*. J. Bacteriol. 195, 867-875 (2013).
