# Supplementary material for: Optimising PHBV biopolymer production in haloarchaea via CRISPRi-mediated redirection of carbon flux
Source: Commun Biol. 2021 Aug 25;4:1007. doi: 10.1038/s42003-021-02541-z (PMC8387396; doi:10.1038/s42003-021-02541-z)
Supplement: Supplementary file 3 — Description of Additional Supplementary Files [file 42003_2021_2541_MOESM3_ESM.pdf]

## Description of Additional Supplementary Files

**File name:** Supplementary Data 1

**Description:** Plasmids used in this study.

**File name**: Supplementary Data 2

**Description:** Primers and mini-CRISPR assay sequences used in this study.

**File name:** Supplementary Data 3

**Description**: Raw data for Fig. 2-6. In total, there are 15 sheets. Each sheet shows the raw data for each individual figure. The sheets are named according to the figure number.
